# Mycobactericidal Effects of Different Regimens Measured by Molecular Bacterial Load Assay among People Treated for Multidrug-Resistant Tuberculosis in Tanzania

**DOI:** 10.1128/JCM.02927-20

**Published:** 2021-03-19

**Authors:** Peter M. Mbelele, Emmanuel A. Mpolya, Elingarami Sauli, Bariki Mtafya, Nyanda E. Ntinginya, Kennedy K. Addo, Katharina Kreppel, Sayoki Mfinanga, Patrick P. J. Phillips, Stephen H. Gillespie, Scott K. Heysell, Wilber Sabiiti, Stellah G. Mpagama

**Affiliations:** a Kibong’oto Infectious Diseases Hospital (KIDH), Siha, Kilimanjaro, Tanzania; b Department of Global Health and Biomedical Sciences, School of Life Sciences and Bioengineering, Nelson Mandela African Institution of Science and Technology (NM-AIST), Arusha, Tanzania; c National Institute for Medical Research, Mbeya Medical Research Centre, Mbeya, Tanzania; d Department of Bacteriology, Noguchi Memorial Institute for Medical Research, University of Ghana, Accra, Ghana; e National Institute for Medical Research, Muhimbili Centre, Dar Es Salaam, Tanzania; f UCSF Center for Tuberculosis, University of San Francisco, San Francisco, California, USA; g School of Medicine, University of St Andrews, St. Andrews, Scotland, United Kingdom; h Division of Infectious Diseases and International Health, University of Virginia, Charlottesville, Virginia, USA; St. Boniface Hospital

**Keywords:** Kibong'oto, Tanzania, MDR-TB treatment regimens, molecular bacterial load assay, multidrug-resistant TB, mycobactericidal effects, *Mycobacterium tuberculosis*, all-oral bedaquiline regimen, injectable aminoglycoside regimen

## Abstract

Rifampin or multidrug-resistant tuberculosis (RR/MDR-TB) treatment has largely transitioned to regimens free of the injectable aminoglycoside component, despite the drug class’ purported bactericidal activity early in treatment. We tested whether Mycobacterium tuberculosis killing rates measured by tuberculosis molecular bacterial load assay (TB-MBLA) in sputa correlate with composition of the RR/MDR-TB regimen. Serial sputa were collected from patients with RR/MDR- and drug-sensitive TB at days 0, 3, 7, and 14, and then monthly for 4 months of anti-TB treatment. TB-MBLA was used to quantify viable M. tuberculosis 16S rRNA in sputum for estimation of colony forming units per ml (eCFU/ml). M. tuberculosis killing rates were compared among regimens using nonlinear-mixed-effects modeling of repeated measures. Thirty-seven patients produced 296 serial sputa and received treatment as follows: 13 patients received an injectable bedaquiline-free reference regimen, 9 received an injectable bedaquiline-containing regimen, 8 received an all-oral bedaquiline-based regimen, and 7 patients were treated for drug-sensitive TB with conventional rifampin/isoniazid/pyrazinamide/ethambutol (RHZE). Compared to the adjusted M. tuberculosis killing of −0.17 (95% confidence interval [CI] −0.23 to −0.12) for the injectable bedaquiline-free reference regimen, the killing rates were −0.62 (95% CI −1.05 to −0.20) log_10_ eCFU/ml for the injectable bedaquiline-containing regimen (*P* = 0.019), −0.35 (95% CI −0.65 to −0.13) log_10_ eCFU/ml for the all-oral bedaquiline-based regimen (*P* = 0.054), and −0.29 (95% CI −0.78 to +0.22) log_10_ eCFU/ml for the RHZE regimen (*P* = 0.332). Thus, M. tuberculosis killing rates from sputa were higher among patients who received bedaquiline but were further improved with the addition of an injectable aminoglycoside.

## Abstract (precis)

Rifampin or multidrug-resistant tuberculosis (RR/MDR-TB) treatment has largely transitioned to regimens free of the injectable aminoglycoside component, despite the drug class’ purported bactericidal activity early in treatment. We tested whether Mycobacterium tuberculosis

## INTRODUCTION

Measurement of pulmonary tuberculosis (PTB) treatment response in areas of endemicity largely depends on sputum smear microscopy (1). While the sputum smear microscopy detection threshold is at least 10^3^ Mycobacterium tuberculosis colony forming units in 1 ml (CFU/ml) of sputum sample, many patients with PTB, such as those with human immunodeficiency virus and AIDS (HIV/AIDS), present with paucibacillary disease and may be unable to produce a good quality sputum for detection of acid-fast bacilli (AFB) (2, 3). Besides, microscopy does not distinguish viable from nonviable M. tuberculosis and therefore does not inform how patients with rifampin- and/or multidrug-resistant (RR/MDR)-TB respond to treatment (3). Patients with RR/MDR-TB are typically monitored for cultured growth in Lowenstein-Jensen (LJ) solid medium or the Mycobacteria Growth Indicator Tube (MGIT) liquid culture system. This culture system is sensitive, with a detection limit of 10 to 100 CFU/ml of sputum, yet it is also prone to contamination and can take up to 8 weeks to determine a definitive positive or negative result, thereby limiting the ability to take appropriate and timely clinical action (4).

The DNA-based methods, such as Xpert MTB/RIF (Cepheid, Sunnyvale, CA, USA) and line probe assays (LPA) like genotype MTBDRplus/sl (Hain Lifescience GmbH, Nehren, Germany) are rapid in detecting M. tuberculosis compared to culture-based methods (5, 6). Nevertheless, DNA of M. tuberculosis can persist in a patient’s sputum for a prolonged time after successful treatment (7, 8). Even if the sputum sample is pretreated with propidium monoazide (Biotium Inc., Hayward, CA, USA), a chemical substance previously known to bind the DNA of dead bacilli, the Xpert MTB/RIF (Cepheid, Sunnyvale, CA, USA) cannot distinguish viable from nonviable DNA of M. tuberculosis (9, 10). Therefore, DNA-based assays are not suitable for monitoring TB treatment response. Fortunately, there is growing evidence that RNA can serve as a surrogate biomarker for microbial viability (11–13), and therefore can be used for monitoring TB treatment response (14, 15). TB molecular bacterial load assay (TB-MBLA) was first reported in 2011 as a biomarker for drug-sensitive TB treatment response and proved a robust measure in different settings (14, 16). TB-MBLA is a real-time quantitative PCR (RT-qPCR) assay that detects and quantifies killing of 16S rRNA from both viable replicating and dormant M. tuberculosis in patient sputum during treatment (14). It is rapid and results are available within 24 h, thereby allowing informed clinical assessment of patient progress (14, 17). TB-MBLA was found to be consistently read as positive for samples with as low as 10 CFU/ml of M. tuberculosis. This bacterial load corresponds to a RT-qPCR quantification cycle (Cq) value of 30, the limit of quantification for positive M. tuberculosis (14). Therefore, TB-MBLA has the potential to replace or complement both smear microscopy and culture for monitoring TB treatment response (14, 16, 18).

Because the treatment is difficult and complex, more that 40% of patients treated for RR/MDR-TB in 2019 worldwide had an unfavorable treatment outcome (19). However, the 10-year RR/MDR-TB treatment success, defined as the total number of patients who achieve microbiological cure and those who complete the treatment regimen, has been consistently above 75% in Tanzania (20). During this decade, the injectable aminoglycoside class of antibiotics, such as kanamycin, has been one of the backbones of RR/MDR-TB treatment regimens (21). Because administering injectable aminoglycoside is not only invasive to patients but is also associated with severe adverse events, including ototoxicity and nephrotoxicity (22, 23), the WHO has endorsed a transition from injectable to all-oral MDR-TB treatment regimens (24). To align with this transition, countries where TB is endemic, including Tanzania, have recently adopted new and repurposed TB medicines, such as bedaquiline, delamanid, and linezolid, and constructed regimens with limited microbiological evidence of effectiveness in patients with RR/MDR-TB. Hence, we deployed TB-MBLA to describe killing of M. tuberculosis in patients receiving RR/MDR-TB and drug-susceptible TB (DS-TB) treatment. We tested the hypothesis that M. tuberculosis killing rates from the sputum, as measured by TB-MBLA, not only correlated with time-to-culture conversion but were dependent upon the composition of the RR/MDR-TB antibiotic regimen.

## MATERIALS AND METHODS

### Patients and ethical considerations.

From August 2018 to December 2019, two populations of patients with TB participated in this study. The primary target population was patients with RR/MDR-TB. A small population of patients with DS-TB was included as a control to inform the reproducibility of previous TB-MBLA study findings (14, 16). Rifampin susceptibility in M. tuberculosis was confirmed using Xpert MTB/RIF (25). Moreover, all patients harbored M. tuberculosis that was deemed susceptible to fluoroquinolones and aminoglycosides by line probe assay (Hain, LifeScience, Germany), the genotype MTBDRsl version 2.0 (26). The study included all patients aged at least 18 years who were able to expectorate and provide quality early morning sputum. Quality sputum was defined by an adequate volume of >5 ml and absence of food particles. No sputum induction was done to patients who were unable to provide quality sputum. Critically ill or moribund patients, as previously defined by Robertson et al. (27), and pregnant women were excluded. Additionally, patients who interrupted treatment were excluded from the final analysis. Prior to any study procedure, all patients signed a witnessed oral or written informed consent. The study was approved by the National Institute for Medical Research (NIMR) in Tanzania (NIMR/HQ/R.8a/Vol. IX/2662). Permission to conduct the study was granted by authorities of the Kibong’oto Infectious Diseases Hospital (KIDH).

### Study design and treatment regimens.

This was a longitudinal cohort study design where each patient was followed for 16 weeks of anti-TB treatment. The treatment regimens for patients with RR/MDR-TB were as follows: (i) an injectable but bedaquiline-free regimen composed of daily dosed kanamycin (15 mg/kg), levofloxacin (750 mg for patients weighing <50 kg and 1,000 mg for those weighing ≥50 kg), pyrazinamide (25 mg/kg), ethionamide (750 mg), and cycloserine (750 mg); (ii) an all-oral based regimen composed of bedaquiline (400 mg daily for 2 weeks, and then 200 mg thrice per week), linezolid (600 mg/day), levofloxacin, pyrazinamide, and ethionamide; (iii) an injectable bedaquiline-containing regimen composed of kanamycin, bedaquiline, levofloxacin, pyrazinamide, and ethionamide; and (iv) a standard fixed-dose combination containing rifampin (150 mg), isoniazid (75 mg), pyrazinamide (400 mg), and ethambutol (275 mg), termed RHZE, for patients with DS-TB. Patients weighing <50 kg received three tablets of RHZE and those weighing ≥50 kg received four tablets of RHZE per day.

### Study setting.

Patients were recruited at Kibong’oto Infectious Diseases Hospital, a national center of excellence for clinical management of drug resistant (DR)-TB located in the Siha district of the Kilimanjaro region in Tanzania (25). TB-MBLA testing was performed at the National Institute for Medical Research, Mbeya Medical Research Centre branch, given that laboratory’s prior experience with the assay.

### Sample size determination.

The numbers of patients required to determine differences in bactericidal activity over time in 4 treatment regimens were calculated as previously reported by Guo et al. (28). We assumed a Spearman correlation of 0.51 and a baseline M. tuberculosis burden of 5.5 log_10_ eCFU/ml, as well as daily M. tuberculosis decline and decay rate of 0.42 and 0.05 log_10_ eCFU/ml, respectively (14, 16). Hence, at least 7 patients were needed per regimen to reach a power of 80% with a two-sided type I error of 5%. Adjusting for least 25% of patients who were likely to be lost to follow-up, not evaluated due to negative microbiological results at baseline, and/or died, a minimum of 37 patients was desirable for sampling and analyzing at the end of 4 months of treatment.

### Sputum collection, processing, and culturing of M. tuberculosis.

One sample of approximately 5 ml of early morning sputum was collected from each patient for laboratory testing at day 0 (baseline) and at days 3, 7, 14, 28, 56, 84, and 112 of anti-TB treatment. Before culturing, sputum was homogenized using a sterile magnetic stirrer at room temperature for 30 min. Then, 1 ml of homogenized sputum was treated using 4 ml of 4 M guanidine thiocyanate (GTC) containing 1 M Tris-HCl (pH 7.5) and 1% (vol/vol) of β-mercaptoethanol, and was frozen at −80°C in order to preserve the M. tuberculosis RNA from degrading. The M. tuberculosis culture was performed on LJ slants from the remaining sputum samples collected at six time points from days 0, 14, 28, 56, 84, and 112 of treatment, as per previous description (29). In brief, sputum was decontaminated by *N*-acetyl-L-cysteine (NALC)-NaOH, and finally resuspended in 1 ml of phosphate buffer. A total of 200 μl of decontaminated sputum was inoculated into two LJ slants and incubated for up to 8 weeks to detect mycobacterial growth. Incubated LJ slants were read on a weekly basis and were deemed negative if there was no growth at week 8.

### RNA extraction and RT-qPCR for TB-MBLA.

M. tuberculosis quantification by TB-MBLA was performed as described by Gillespie et al. (30). In summary, M. tuberculosis RNA in 1 ml of homogenized sputum preserved in 4 ml of guanidine thiocyanate (GTC) at −80°C was extracted using the RNA pro kit (FastRNA Pro BlueKit; MP Biomedical, CA, USA) as instructed by the manufacturer. The extract was treated with DNase I enzyme (TURBO DNA-free kit; Ambion, CA, USA) to remove DNA from the dead cells. The M. tuberculosis 16S rRNA, a biomarker for viable cells, was amplified and quantified by RT-qPCR using specific primers and probes. The Cq was translated to bacterial load (estimated CFU per ml [eCFU/ml]) using a standard curve on a Rotor gene Q 5plex platform (Qiagen). The cutoff for TB-MBLA positivity was a 30 Cq value that corresponds to 1.0 log_10_ eCFU/ml, beyond which the test was considered negative (16, 30).

### Statistical analysis.

Data were recorded in a clinical case report form, entered, and cleaned before statistical analysis. Patients who completed 8 treatment visits and had positive pretreatment TB-MBLA results were analyzed and visualized in R softward version 4.0.2 (http://www.R-project.org). Continuous variables, such as age, body mass index (BMI) in kg/m^2^, and time to TB-MBLA negativity were described by the median with the 25^th^ and 75^th^ interquartile range (IQR), and were compared across different regimens using a Kruskal-Wallis test. Accordingly, proportions for HIV status, gender, cavitary lung disease on chest, and previous TB treatment were compared across different regimens using a chi-square or Fisher’s exact test. Using baseline bacterial load, chest cavity, HIV, silicosis, and gender as fixed effects, the rate of M. tuberculosis killing (log_10_eCFU/ml) was fitted on quartic polynomial nonlinear mixed effects (NLME) for repeated measures as previously described (31, 32). Individual patients were accounted for random effect. A model was reliably selected if it had low Akaike information criterion but high intraclass correlation coefficient (Table 1). The mean difference in M. tuberculosis load, due to two different regimens received by patients at the end of 4 months of treatment, was compared using one-way analysis of variance (ANOVA) and Tukey’s test for repeated measures (33). An injectable regimen without bedaquiline was used as a reference regimen. The median time to TB-MBLA and culture conversion to negative was estimated using the Kaplan-Meier method, and was compared across different regimens using a log rank test (34). Cox proportional hazards regression models were used to estimate the hazard ratios (HR) for M. tuberculosis killing, and was adjusted for the effects of HIV, baseline bacillary load, cavitary disease, silicosis, gender, history of treatment for drug-sensitive TB, and clearance rate. We computed an overall mean M. tuberculosis load of 4.0 log_10_ eCFU/ml, and it was used to categorize a patient’s bacterial load as “high bacterial load” versus “low bacterial load” depending on whether patients had detectable M. tuberculosis above or below this mean, respectively. A *P* value of <0.05 was considered significant. A 95% confidence interval (CI) of the mean clearance rate and HR was included.

**TABLE 1:** Fitting and selection of a reliable polynomial nonlinear mixed effects model for repeated measures^*a*^

Polynomial models (degree)^*b*^	Intercepts (log_10_ eCFU/ml)	ICC	SD	AIC	Likelihood ratio test	*P* value
Nonpolynomial (model 1)	3.00	0.54	0.81	722.89	1 versus 2	< 0.001
Quadratic (model 2)	2.99	0.63	0.67	634.63		
Cubic (model 3)	3.00	0.65	0.63	611.59	2 versus 3	< 0.001
Quartic (model 4)	3.20	0.67	0.61	592.7	3 versus 4	< 0.001
Quintic (model 5)	2.89	0.68	0.60	588.58	4 versus 5	0.020

a ICC, intraclass correlation coefficient; SD, standard deviation; AIC, Akaike information criterion.

b Model 4 had the lowest AIC and within variability (SD) but high ICC values, the key selection criteria for a reliable model, and hence it was used to model M. tuberculosis killing rates.

## RESULTS

### Population.

**FIG 1:**
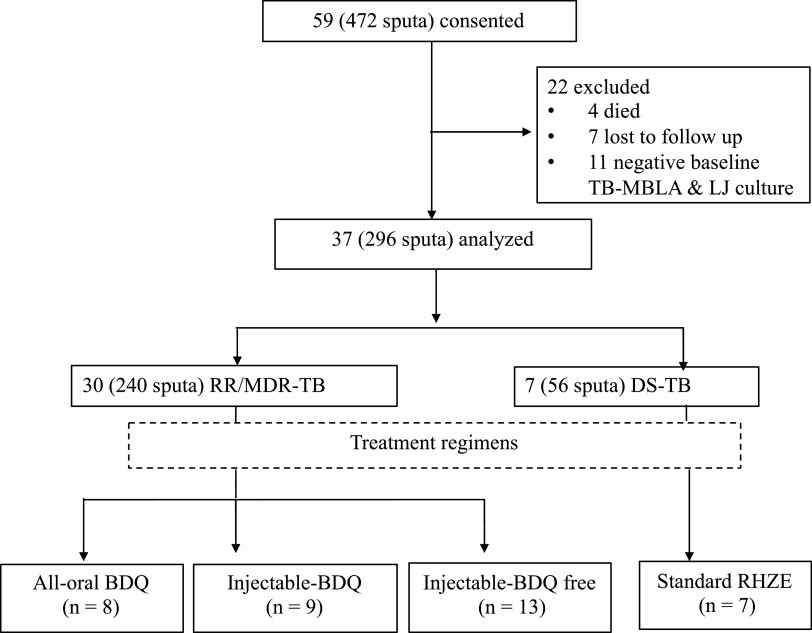
Recruitment and patient distribution into different treatment regimens. Patients with drug-sensitive (DS) and rifampin/multidrug-resistant (RR/MDR)-TB were recruited and treated using different anti-TB treatment regimens. Regimens included (i) standard RHZE composed of rifampin, isoniazid, pyrazinamide, and ethambutol; (ii) injectable bedaquiline (BDQ)-free regimen composed of kanamycin (KAN), levofloxacin (LFX), pyrazinamide (PZA), ethionamide (ETH), and cycloserine (CS); (iii) injectable BDQ-containing regimen composed of KAN, BDQ, LFX, PZA, and ETH; and (iv) all-oral BDQ regimen contained BDQ, LFX, linezolid (LZD), PZA, and ETH. Among other criteria, 11 out of 59 patients recruited had negative TB molecular bacterial load assay (TB-MBLA) results at baseline and were excluded from the final analysis.

Of 59 patients enrolled, 37 patients produced a total of 296 serial sputa for final analysis. Reasons for exclusion and patient’s distribution are outlined in Fig. 1. Among 296 serial sputa analyzed, 104 sputa came from 13 patients who received an injectable but bedaquiline-free regimen, 72 sputa were from 9 patients who received an injectable bedaquiline-containing regimen, 64 from 8 patients who received an all-oral bedaquiline-based regimen, and 56 sputa from 7 patients who were treated for drug-sensitive TB with conventional RHZE. Clinical and demographic parameters are presented in Table 2. Twenty-seven (73%) out of 37 patients were male. Their median (IQR) age was 37 (32 to 49) years. Patients who received standard RHZE treatment were younger than those who received RR/MDR-TB treatment regimens (*P* = 0.038). Also, 11 (30%) patients were living with HIV with a CD4 T cell count of 208 (95% CI 144 to 272) cells/μl. More patients with HIV received an all-oral than injectable-based treatment regimen (*P* = 0.001).

**TABLE 2:** Socio-demographic and clinical characteristics of patients per treatment regimen^*a*^

Variable	All	RHZE (*n* = 7)	Injectable ± BDQ (*n* = 21)	All-oral BDQ (*n* = 9)	*P* value
Median age (IQR)	37 (32–49)	30 (29–33)	42 (34–54)	36 (33–44)	0.038
Male (%)	27 (73)	4 (57)	18 (86)	5 (56)	0.125
Chest cavity, *n* (%)	29 (78)	7 (100)	14 (67)	8 (89)	0.163
Probable TB, *n* (%)	34 (92)	7 (100)	18 (86)	9 (100)	0.568
HIV positive, *n* (%)	11 (20)	0 (0)	3 (14)	8 (89)	0.001
TB/Silicosis, *n* (%)	7 (19)	1 (14)	4 (19)	2 (22)	0.731
Malnourished, *n* (%)	22 (59)	4 (57)	11 (52)	7 (78)	0.432
Retreatment, *n* (%)	23 (62)	5 (71)	14 (67)	4 (44)	0.528
Median BMI (IQR)	18 (15–19)	17 (15–20)	18 (16–20)	17 (15–18)	0.301
Median days spent before care (IQR)	84 (60–196)	85 (68–93)	84 (56–196)	88 (68–365)	0.778

a BDQ, bedaquiline; BMI, body mass index; injectable ± BDQ, kanamycin with or without BDQ; IQR, interquartile range. Probable TB was defined as the presence of radiological changes, including cavity, infiltrates, nodules, hilar lymphadenopathy, and aortopulmonary window adenopathy on chest radiograph. *P* value was computed to compare RHZE, injectable ± BDQ, and kanamycin with or without BDQ regimens.

### Mycobactericidal activities of different regimens over time.

**FIG 2:**
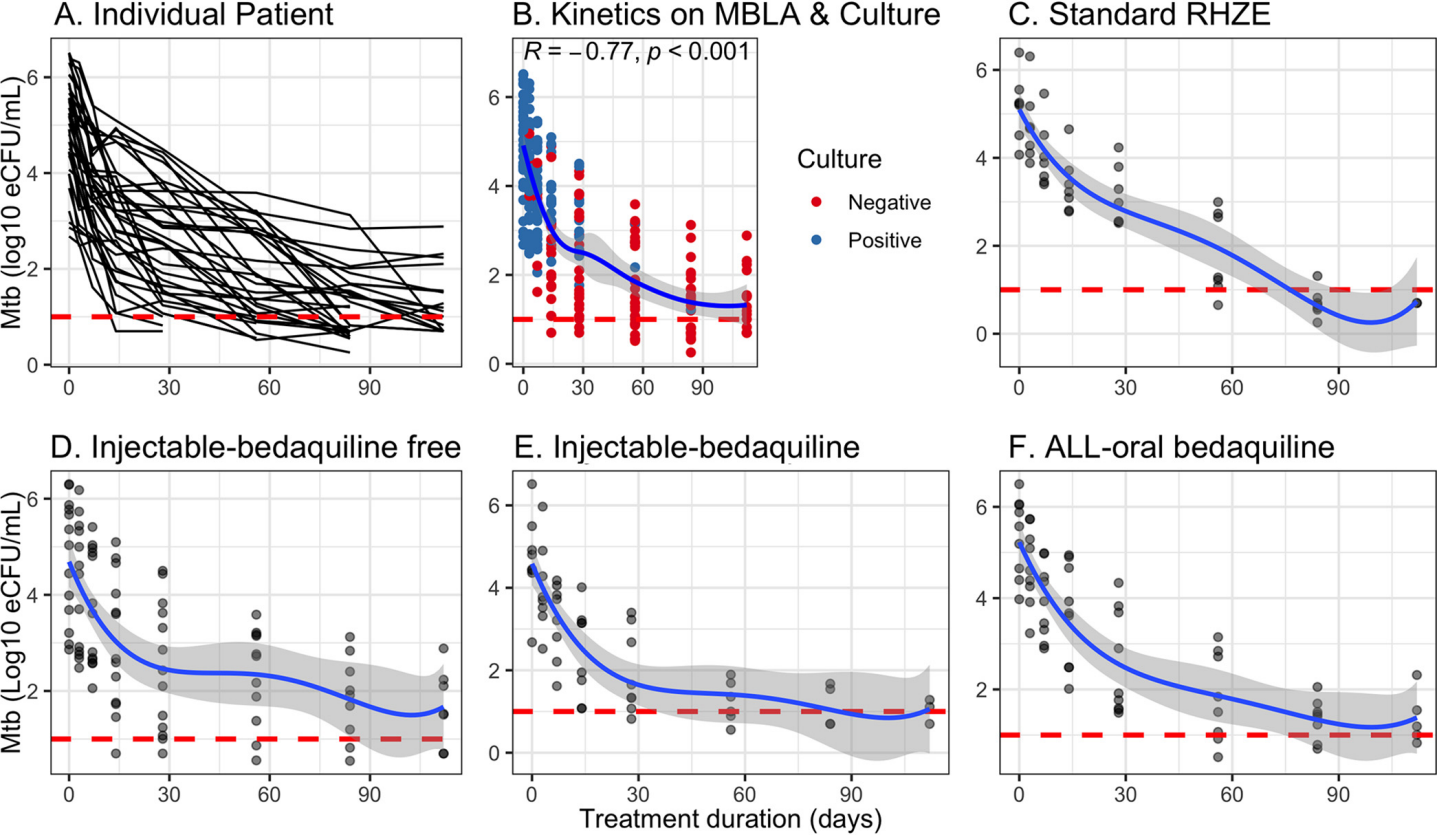
M. tuberculosis killing during the first 4 months of anti-TB treatment. The plots show M. tuberculosis (Mtb) kinetics as measured by TB-MBLA during treatment with different anti-TB regimens. The dotted line is the cutoff value for a positive TB-MBLA test. (A) Overall time-dependent decline of M. tuberculosis load in estimated CFU per 1 ml (eCFU/ml) of sputum between patients as measured by TB-MBLA. (B) Delineation of this decline as measure by both TB-MBLA and Lowenstein-Jensen culture. Overall, bacterial load at baseline has a strong positive correlation with median time to sputum conversion to negative by both TB-MBLA and culture. Patients with higher bacterial load at baseline had a later culture conversion to negative than those with lower baseline loads. (C to F) M. tuberculosis decline in eCFU/ml among patients treated with standard RHZE (C); injectable bedaquiline-free regimen containing kanamycin (KAN), levofloxacin (LFX), pyrazinamide (PZA), ethionamide (ETH), and cycloserine (CS) (D); injectable bedaquiline-containing regimen was composed of KAN, bedaquiline (BDQ), LFX, PZA and ETH (E); and an all-oral bedaquiline regimen containing BDQ, LFX, linezolid (LZD), PZA, and ETH (F).

**FIG 3:**
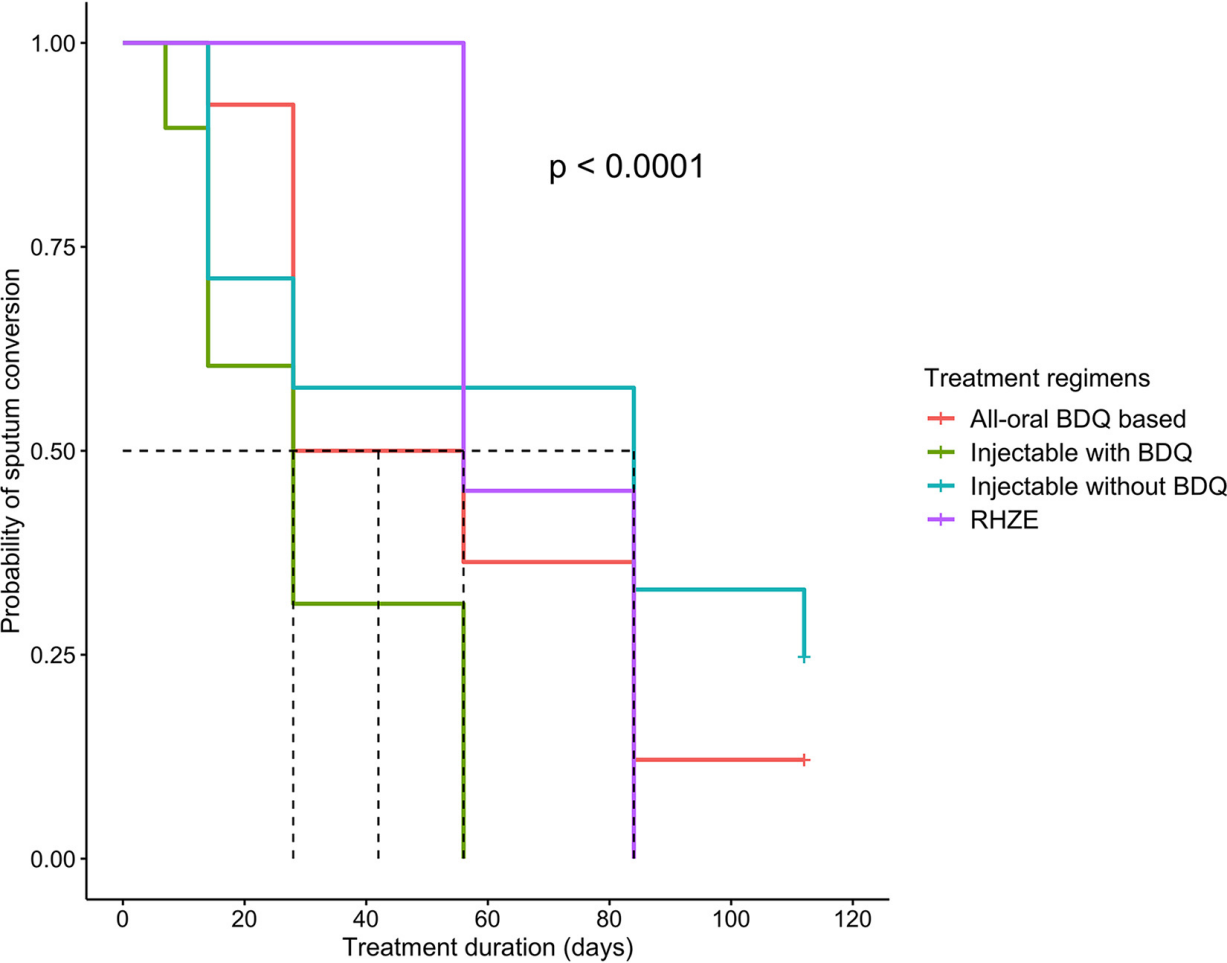
Kaplan-Meier curves showing median time to M. tuberculosis killing in patient sputum per treatment regimen. The dotted lines denote the median time to TB-MBLA conversion from positive to negative. Bedaquiline-containing regimens had short median time to TB-MBLA conversion to negative compared to the injectable but bedaquiline-free regimen containing kanamycin (KAN), levofloxacin (LFX), pyrazinamide (PZA), ethionamide (ETH), and cycloserine (CS). Injectable bedaquiline-containing regimen was composed of KAN, bedaquiline (BDQ), LFX, PZA, and ETH. The all-oral bedaquiline regimen was composed of BDQ, LFX, linezolid (LZD), PZA, and ETH. Standard RHZE was composed of rifampin, isoniazid, PZA, and ethambutol.

The M. tuberculosis load measured by TB-MBLA and culturing in Fig. 2 decreased significantly over time (R = −0.77, *P* < 0.001). The mean M. tuberculosis load in log_10_ eCFU/ml (95% CI) was reduced from 5.19 (4.40 to 5.78) at baseline to 3.10 (2.70 to 3.50) at day 14, then to 2.52 (2.13 to 2.90) at day 28, 1.88 (1.53 to 2.22) at day 56, and <1.36 (1.03 to 1.70) at day 84 through 112 of treatment. The overall mean daily M. tuberculosis killing was −0.24 (95% CI −0.39 to −0.08) log_10_ eCFU/ml, and it varied with treatment regimen (Table 3, *P* < 0.001). An injectable bedaquiline-containing regimen had the highest mean M. tuberculosis killing rate, followed by an all-oral bedaquiline-based regimen compared to the injectable but bedaquiline-free reference regimen (Table 3, *P* = 0.019). Kanamycin-containing regimens in Fig. 3 had rapid bactericidal activity at day 14, but this was not translated into long-term bactericidal effect (*P* < 0.001). An all-oral bedaquiline-based regimen had a sharp decline after day 28.

**TABLE 3:** Mean daily M. tuberculosis killing rates (log_10_ eCFU/ml) and corresponding burden at day 0 (baseline) and day 112 of treatment

Treatment regimens^*c*^	Mean M. tuberculosis killing rates				Mean (95% CI) M. tuberculosis load	
	Unadjusted model for covariates		Adjusted model for covariates			
	Rates (95% CI)	*P* value	Rates (95% CI)	*P* value	Day 0 (baseline)^*a*^	Day 112^*b*^
1. Reference (injectable BDQ-free)	−0.18 (−0.27 to −0.08)		−0.17 (−0.23 to −0.12)		4.73 (4.13–5.32)	2.77 (2.51–3.04)
2. Injectable with bedaquiline	−0.48 (−1.25 to +0.28)	0.239	−0.62 (−1.05 to −0.20)	0.019	4.63 (3.95–5.47)	2.08 (1.81–2.36)
3. All-oral bedaquiline	−0.26 (−0.48 to +1.00)	0.507	−0.35 (−0.65 to −0.13)	0.054	5.36 (4.65–6.08)	2.47 (2.20–2.74)
4. Standard RHZE	−0.23 (−0.57 to +1.02)	0.593	−0.29 (−0.78 to +0.22)	0.332	5.17 (4.36–5.99)	2.51 (2.18–2.85)

a Baseline mean M. tuberculosis load in all regimens were comparable (ANOVA, *P* = 0.453).

b *P* values for mean differences in M. tuberculosis load for regimens by pairwise comparison at day 112 were as follows: regimens 1 and 2, *P* < 0.001; regimens 2 and 3, *P* = 0.031; regimens 1 and 3, *P* = 0.077; and regimens 2 and 4, *P* = 0.040.

c Reference regimen was the injectable bedaquiline (BDQ)-free regimen composed of kanamycin (KAN), levofloxacin (LFX), pyrazinamide (PZA), ethionamide (ETH), and cycloserine (CS). Injectable bedaquiline regimen was composed of KAN, BDQ, LFX, PZA, and ETH. All-oral bedaquiline regimen contained BDQ, LFX, linezolid (LZD), PZA, and ETH. RHZE contained rifampin, isoniazid, PZA, and ethambutol (E). Covariates adjusted included baseline bacterial load, cavity, gender, HIV, and silicosis; M. tuberculosis killing rates varied among regimens.

### Median time to M. tuberculosis killing.

**FIG 4:**
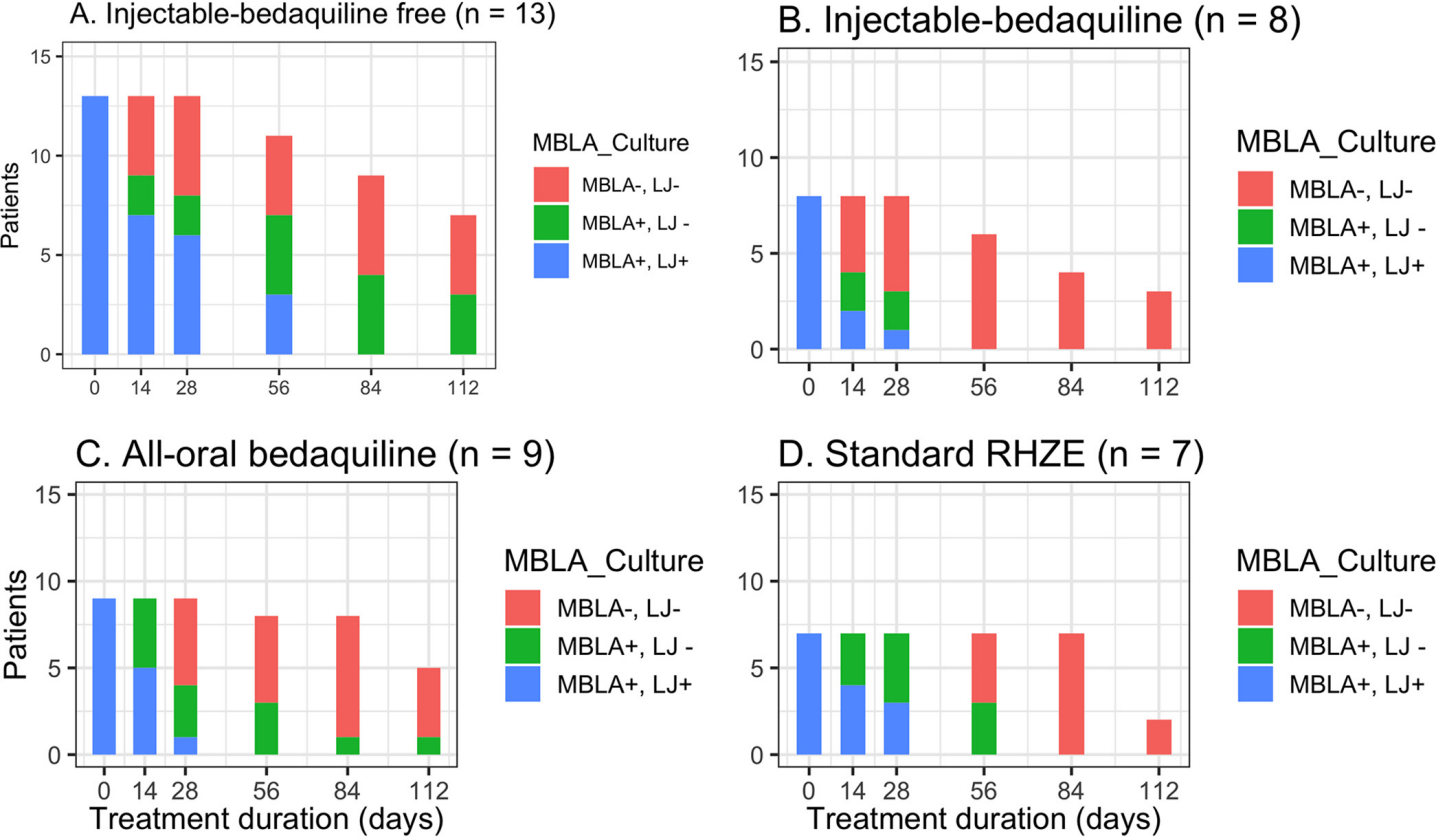
Number of patients who converted to negative by TB-MBLA and Lowenstein-Jensen culture during the first 4 months of treatment with different anti-TB regimens. The overall sputum conversion from positive to negative TB-MBLA and LJ culture results had the same trend in four different regimens. At recruitment (day 0), all 37 patients had positive results for TB by TB-MBLA and culture (MBLA+, LJ+). Both TB-MBLA and culture tests were negative (MBLA−, LJ−) at days 56 and 84, respectively, in all patients on either injectable plus bedaquiline (B) or standard RHZE (D) composed of rifampin, isoniazid, PZA, and ethambutol. A total of 3 patients who received the injectable bedaquiline-free regimen (A), together with 1 patient on the all-oral bedaquiline regimen (C), remained TB-MBLA positive but culture negative (MBLA+, LJ−).

There was strong positive correlation in time to sputum conversion between TB-MBLA and culture (r = 0.46 [95% CI 0.36 to 0.55]; *P* < 0.001). The overall median time to sputum TB-MBLA conversion to negative was 56 (IQR 28 to 84) days. The median times to TB-MBLA conversion to negative were 28, 42, and 84 days among patients on injectable bedaquiline, an all-oral bedaquiline-based regimen, and injectable but bedaquiline-free regimens, respectively. Irrespective of treatment regimen, 92% (34/37) of patients had negative culture results compared to 65% (24/37) of negative TB-MBLA at day 56 (*P* = 0.037). The number of patients who converted to sputum negative by culture and TB-MBLA per treatment regimen is shown in Fig. 4. Among 13 patients who received the injectable but bedaquiline-free regimen, 2 and 7 of them remained culture and TB-MBLA positive, respectively, whereas all 8 patients who received injectable bedaquiline-containing regimens had negative LJ culture and TB-MBLA at day 56 (Fig. 4A to D). Favorably, all patients on injectable bedaquiline for MDR-TB and standard RHZE regimen for DS-TB had negative TB-MBLA at days 56 and 84, respectively. Compared to 31% (4/13) of patients who received an injectable but bedaquiline-free regimen, only 11% (1/9) of those who received an all-oral bedaquiline-containing regimen remained positive TB-MBLA but negative LJ culture at day 112 of treatment (Fig. 4A and B versus Fig.4E and F; *P* = 0.283).

### Hazard ratios of M. tuberculosis killing.

The overall mean M. tuberculosis load log_10_ eCFU/ml at baseline was 5.19 (95% CI 4.40 to 5.78), and was similar in all patients treated with any of the 4 regimens (Table 3, *P* = 0.453). The mean M. tuberculosis load (log_10_ eCFU/ml) among female patients was 5.6 (95% CI 5.0 to 6.2) log_10_ eCFU/ml compared to 4.7 (95% CI 4.3 to 5.2) log_10_ eCFU/ml among male patients (*P* = 0.017). Patients with chest cavity had mean M. tuberculosis load of 5.26 (95% CI 4.45 to 5.87) compared to 4.40 (95% CI 3.91 to 4.75) log_10_ eCFU/ml in those without cavity (*P* = 0.080). Adjusting for bacterial load, initial killing rate, silicosis, chest cavity, HIV status, and gender, the hazard ratios (HR) for M. tuberculosis killing were 12.37 (95% CI 2.87 to 53.30; *P* = 0.001) and 14.31 (95% CI 3.49 to 58.65; *P* < 0.001) for patients who received an all-oral bedaquiline versus injectable bedaquiline-containing regimens, respectively (Table 4). Bacterial load at baseline strongly correlated positively with median time to sputum conversion to negative measured by both TB-MBLA and culture (*r* = 0.48 [95% CI 0.18 to 0.69]; *P* = 0.003). High M. tuberculosis load and TB with silicosis were independent predictors of slow M. tuberculosis killing compared to low M. tuberculosis load and TB without silicosis (Table 4, *P* ≤ 0.033).

**TABLE 4:** Hazard ratio (HR) of M. tuberculosis killing in a Cox proportion-hazard model

Predictor variable^*a*^	Unadjusted model		Adjusted model	
	HR (95% CI)	*P* value	HR (95% CI)	*P* value
Male gender	0.86 (0.40–1.85)	0.705	2.44 (0.82–7.24)	0.109
TB/silicosis	0.20 (0.10–0.88)	0.028	0.12 (0.03–0.49)	0.003
TB/HIV	2.26 (1.07–4.77)	0.033	0.88 (0.31–2.50)	0.813
Cavitary disease	0.38 (0.17–0.86)	0.021	0.85 (0.17–2.70)	0.790
Positive chest x-ray	0.57 (0.17–1.88)	0.354	0.23 (0.03–1.62)	0.790
High M. tuberculosis load	0.72 (0.54–0.97)	0.033	0.26 (0.13–0.54)	<0.001
Retreatment	1.02 (0.51–2.05)	0.958	0.59 (0.24–1.44)	0.248
All-oral bedaquiline	1.58 (0.61–4.04)	0.344	12.37 (2.87–53.30)	0.001
Injectable-bedaquiline	4.63 (1.64–13.09)	0.004	14.31 (3.49–58.65)	<0.001
Standard RHZE	1.43 (0.53–3.89)	0.482	3.25 (0.90–11.73)	0.072
High initial M. tuberculosis killing rate	5.96 (2.03–17.48)	0.009	4.81 (1.39–16.65)	0.013

a All-oral bedaquiline regimen was composed of bedaquiline (BDQ), levofloxacin (LFX), linezolid (LZD), pyrazinamide (PZA), and ethionamide (ETH). Injectable bedaquiline is a modified regimen composed of kanamycin (KAN), BDQ, LFX, PZA, and ETH. Standard RHZE included rifampin (H), isoniazid (H), PZA, and ethambutol (E).

## DISCUSSION

This study shows for the first time to our knowledge that the killing rates of M. tuberculosis in patients treated for RR/MDR-TB, as well as those with concomitant TB with silicosis, varies with treatment regimens. As measured by TB-MBLA, M. tuberculosis decreased significantly over time on treatment, and this kinetic correlated with what was observed using LJ culture medium. For decades, culture has been used as a routine microbiological tool for monitoring drug-resistant TB treatment response (15, 35), but in many settings with endemic TB, culture is unavailable or limited to specialized centers. Importantly, culture results can take up to 8 weeks from the time of sputum collection, which delays patient care if a treatment decision is made based on a result from a specimen collected 2 months earlier. Given the continued decentralization of RR/MDR-TB services in Tanzania and elsewhere, monitoring treatment response in laboratories capable of performing qPCR, such as with Xpert MTB/RIF, will allow laboratory assays to impact treatment decisions closer to the point-of-care. Therefore, this study in RR/MDR-TB complements the growing evidence supporting the application of TB-MBLA in routine clinical management (14, 16, 36).

Interestingly, our findings suggest that bactericidal activity at day 14 may not be a suitable predictor of the long-term efficacy of a regimen, particularly when that regimen contains bedaquiline. In this cohort at day 14, more than 75% of people had a positive TB-MBLA and more than half had a positive culture result. However, between 14 and 56 days we observed substantial M. tuberculosis killing in those treated with a bedaquiline-containing regimens, suggesting that evaluation of bactericidal activity be performed later, such as at day 56, for modern RR/MDR-TB regimens. Using culture, one previous phase 2b clinical trial reported high bactericidal activity of a bedaquiline-containing regimen in patients with DS- and RR/MDR-TB (37). However, detectable M. tuberculosis beyond day 56 in our study supports this trial’s argument that day 56 is unreliable as an indicator of a regimen’s ability to either predict long-term treatment outcomes or shorten treatment duration (37). This further raises the question of whether TB-MBLA may in fact be a superior predictor to culture.

Another important finding from this study of TB-MBLA is that M. tuberculosis killing kinetics were regimen dependent. Overall, there was rapid and prominent killing of M. tuberculosis at day 14 for patients who received kanamycin regardless of receipt of bedaquiline. However, superior activity of kanamycin-containing regimens at day 14 had no long-term bactericidal effect. As a result, 3 patients on the injectable but bedaquiline-free regimen remained positive by TB-MBLA but negative by LJ culture after 4 months of treatment. On the other hand, patients who received an all-oral bedaquiline-containing regimen achieved these rates of killing at or after 1 month of treatment. This observation concurs with previous reports that the bactericidal activity of bedaquiline in MDR-TB is delayed at the beginning, but accelerates later in therapy (38). Usually, recovery of M. tuberculosis by TB-MBLA correlates better with Mycobacteria Growth Indicator Tube (MGIT) liquid culture than with LJ solid culture, which may partially explain the discrepancy between the two tests at month 4 of treatment (16). This argument supports previous findings that culturing M. tuberculosis on LJ recovers a lower yield than in MGIT liquid culture (39). Nonetheless, our findings, as measured by TB-MBLA, fit with the pharmacodynamical understanding that kanamycin and other aminoglycoside/polypeptides that are active against mycobacteria will primarily exert their effect against those extracellular organisms that are rapidly dividing, and these may be more abundant early in the treatment course (40, 41).

The shorter overall time to sputum conversion to negative, as measured by TB-MBLA and conventional culture, for all patients who received bedaquiline regardless of kanamycin further supports the argument that bedaquiline should be a cornerstone of regimens designed to shorten the duration of MDR-TB treatment (42). The conventional injectable but bedaquiline-free regimen has been in practice for decades, even though more than 40% of patients treated with this regimen had unfavorable outcomes in settings where TB is endemic (43). Aminoglycosides such as kanamycin are no longer part of the current MDR-TB treatment regimens, not due to lack of bactericidal activity, as our data would suggest the contrary in the early treatment period, but rather because of the significant toxicity and patient intolerance that leads to treatment interruption (24, 44). From a microbiological perspective alone, as demonstrated in this study and others, such as Mpagama et al. (45), and also in a more patient-centered approach, our results demonstrate the potential importance of finding more tolerable substitutes for kanamycin that can match the early bactericidal effect.

The main strength of this study is that we utilized TB-MBLA to model killing rates among patients with RR/MDR-TB and those with TB/silicosis. We have shown that patients with TB/silicosis had slower M. tuberculosis killing rates by TB-MBLA compared to those with TB and without silicosis. This low rate of killing could partially be attributed to the underlying pulmonary pathophysiology, which can include progressive massive fibrosis (46, 47) and a blunted local host immune response to M. tuberculosis infection (46). We observed a similarly lower rate of M. tuberculosis killing among patients with RR/MDR-TB who had high initial bacterial load, which is consistent with previous studies of TB-MBLA kinetics from patients with drug-sensitive TB (14, 16, 36). In this study, approximately 1 and 4 out of 10 patients had, respectively, positive LJ culture and positive TB-MBLA at day 56. This supports the previous argument that TB-MBLA is more sensitive compared to agar-based Loewenstein-Jensen culture, in which the M. tuberculosis population gets lost due to contamination at later time points (18). Limitations of the study include the timing of endpoints, which were limited to 4 months, such that predicting long-term treatment success was beyond the scope of this study. Nevertheless, modeling M. tuberculosis killing for 4 months as we accomplished here has been used as a marker for treatment failure and relapse in several observational studies (35, 48), and exceeds the duration of monitoring used in other trials of RR/MDR-TB regimens that have employed conventional culture-based techniques (37). Additionally, this study had no control over the treatment regimens prescribed. However, given the feasibility of TB-MBLA and the comparability of this study’s findings to prior studies with TB-MBLA in drug-susceptible TB (16), we plan to apply TB-MBLA systematically within an ongoing operational research protocol for injectable-free RR/MDR-TB treatment in Tanzania that employs standardized regimens over various treatment durations. Lastly, because of the small number of patients per treatment regimen, these findings should be cautiously inferred to other RR/MDR-TB populations. Nevertheless, a longitudinal cohort design in this study allowed control of variabilities between patients, as well as intrapatient tracking of each regimen’s bactericidal activities over time (28, 49).

In conclusion, patients who received bedaquiline-containing regimens exhibited higher M. tuberculosis killing rates and had shorter time to sputum TB-MBLA and culture conversion to negative. While both kanamycin-containing regimens had superior bactericidal activity during the first 2 weeks of RR/MDR-TB treatment, the addition of bedaquiline allowed for improved killing after 1 month of therapy. Together, these findings provide insight into formulating optimal all-oral bedaquiline-containing regimens with the best potential to shorten duration of MDR-TB treatment (37, 44, 50). Given that TB-MBLA does not require laboratory procedures associated with culture and the prolonged time to receive a culture-based result, we envision it can be used to make regimen adjustments in the presence of anti-TB drug susceptibility testing results.
